# Supplementary material for: Host-Induced Gene Silencing of the Aspergillus flavus O-Methyl Transferase Gene Enhanced Maize Aflatoxin Resistance
Source: Toxins (Basel). 2024 Dec 27;17(1):8. doi: 10.3390/toxins17010008 (PMC11769010; doi:10.3390/toxins17010008)
Supplement: Supplementary file 1 [file toxins-17-00008-s001.zip › toxins-3315291-supplementary.pdf]

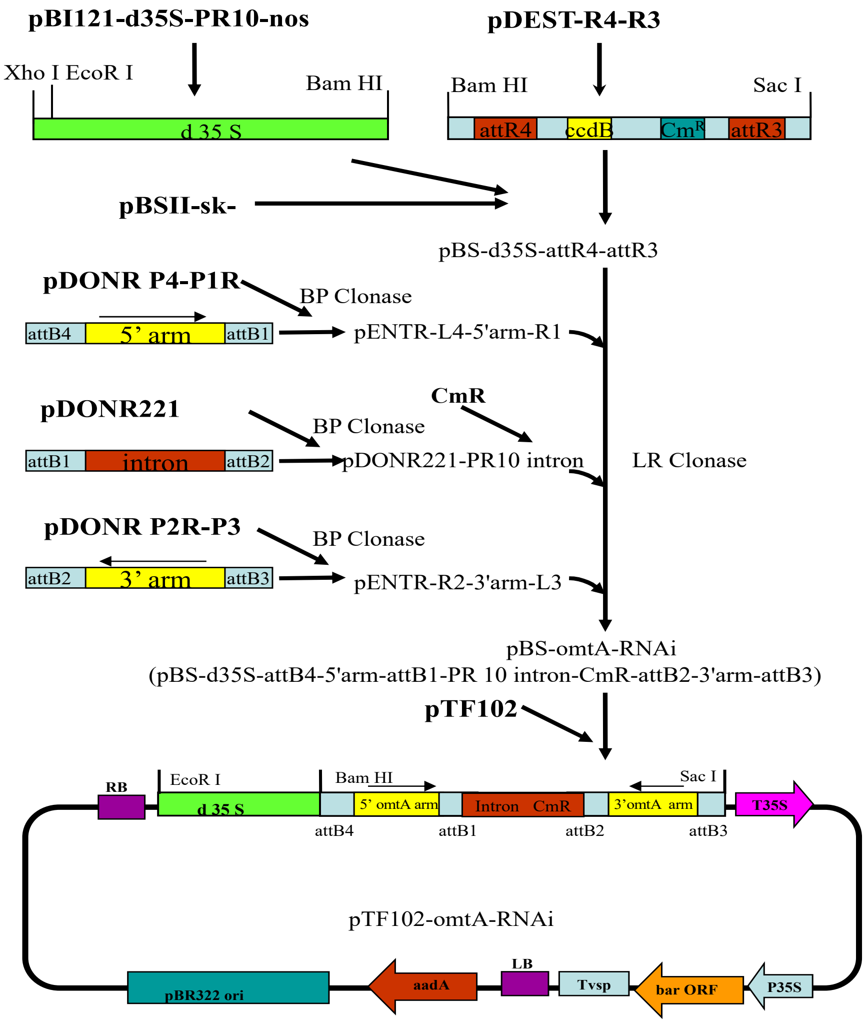

Figure S1. Construction scheme of gene silencing vector. The pBS- d35S- R4-R3 vector containing a double 35S promoter, followed by an attR4-ccdB-CmR-attR3 cassette amplified from pDEST^{TM} R4-R3 (Invitrogen, Carlsbad, CA) in the pBluescript SK- was constructed in an earlier study. The DNA regions corresponding to the *omtA* 5 ′arm and 3′arm were amplified by PCR with primers containing unique homologous recombination sites cloned into their corresponding entry vectors. They were recombined with pDONR221-PR 10-intron-CmR containing a chloramphenicol resistance gene (CmR) selection marker in the middle of the PR10 intron through the LR clonase reaction to assemble the RNAi cassette into the pBluescript vector to produce the pBS-d35S-attB4-5′arm- attB1-PR10 intron-CmR-attB2-3′arm-attB3 vector (named pBS-II-OmtA-RNAi). The RNAi cassette was then cloned into the pTF102 vector through ligation to produce the final pTF102-omtA-RNAi vector. This figure was modified from Chen et al., (2010).

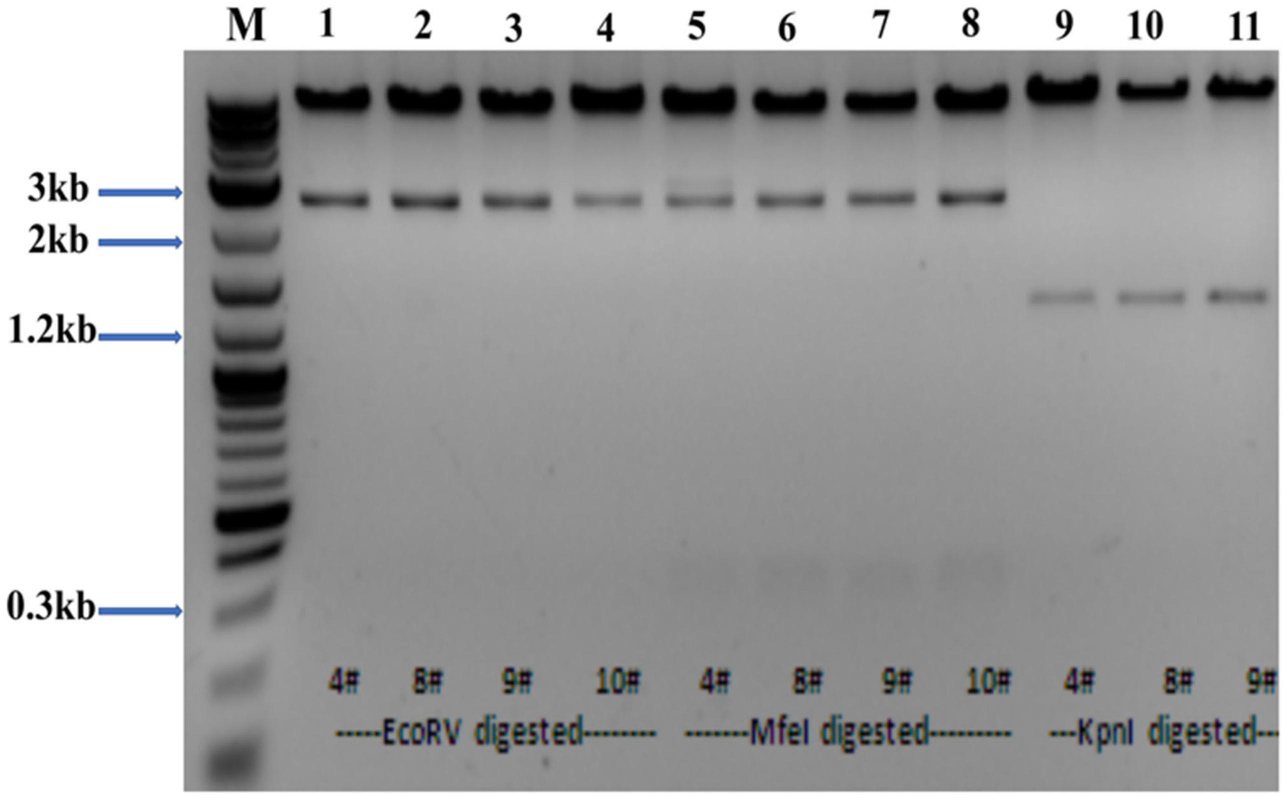

Figure S2. A. Restriction digestion of the pTF102-OmtA-RNAi construct to confirm its correct assembly. Lanes 1-4 were four independent clones digested with EcoR V, which resulted in the expected sizes of 2447 and 9085 bp; and lanes 5-8 digested with Mfe I, which resulted in the expected sizes of 229, 2447, and 8786 bp and lanes 9-11 digested with Kpn I resulting in the expected sizes of 1328 and 10204 bp.

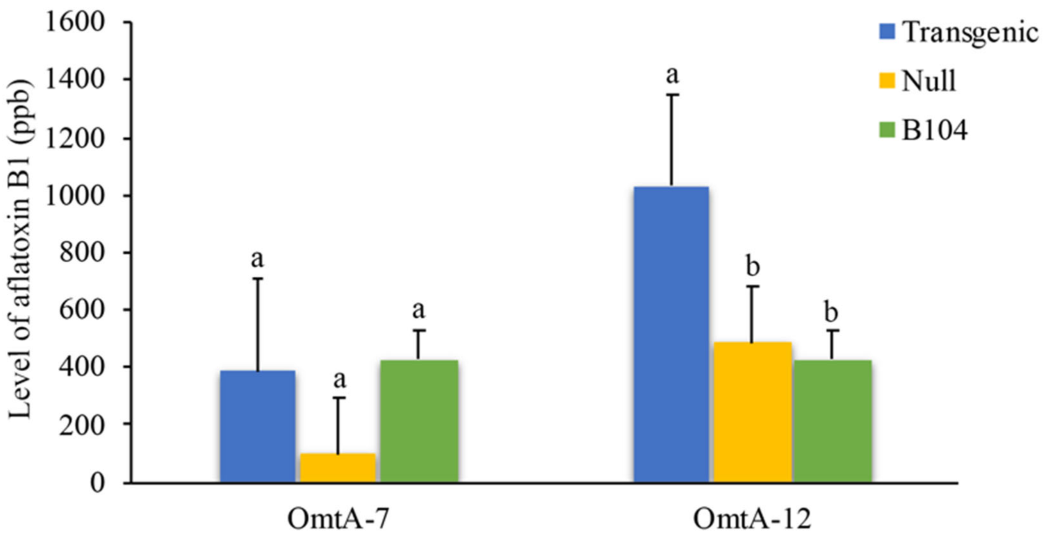

Figure S3. Aflatoxin production in Omt-A-7 and Omt-A-12 transgenic lines and null controls at T4 generation under field condition in 2017. Three to four replicates per ear with 5-10 ears from each line were inoculated using 4 x 10^6 conidia/mL. Bars labeled with the same letters are not significantly different at P≤0.05. In 2017, OmtA-7 and OmtA-12 were selected for further evaluation for resistance to aflatoxin. However, the 2017 field trial suffered severe *Fusarium spp.* infection and the aflatoxin values were generally too low. There was no significant difference between OmtA-7 homozygous transgenic when compared with null lines (P=0.49) and OmtA-12 homozygous transgenic when compared with null lines (P=0.65). Analysis was carried out using 12 ears of OmtA-7 homozygous, 11 of OmtA-7 null, 9 ears of OmtA-12 homozygous, 11 ears of OmtA-12 null and 5 ears of the B104 control with four samples from each ear. Additional field trials were conducted in the following years in order to reliably assess the changes in aflatoxin resistance in these lines.

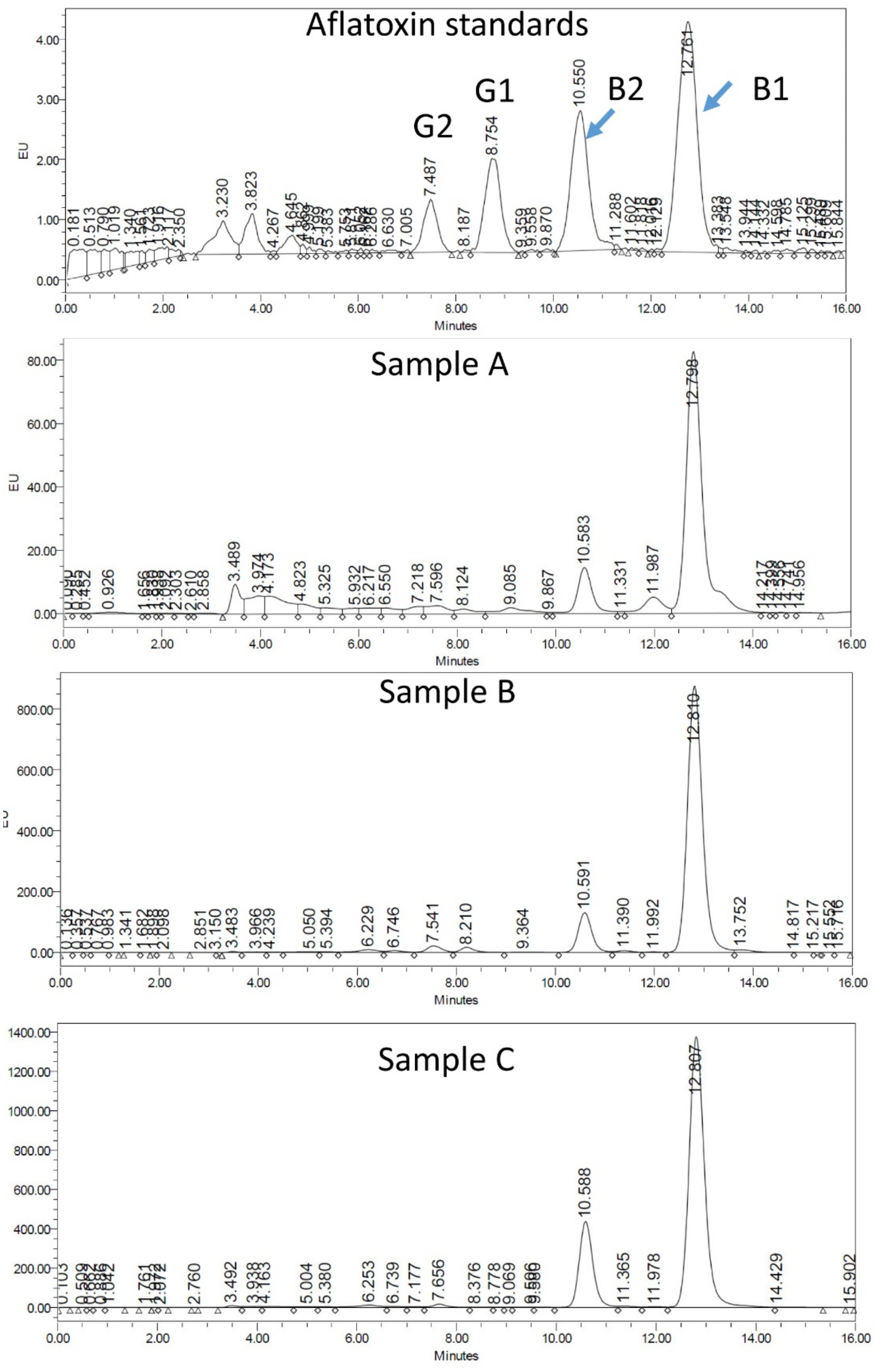

Figure S4. HPLC chromatogram showing the peaks of aflatoxin B1 in standards and in different samples. The retention time is indicated on top of the peaks to confirm the positive peak identification.

Table S1. Aflatoxin accumulation in the seeds of different T1 OmtA transgenic lines received from ISU

Event		Number of kernels	Mean aflatoxin level (ppb)	*P*-value
	Null	11	9,141	
OmtA-6	Transgenic	14	7,242	0.6710
	Null	11	40,862	
OmtA-7	Transgenic	13	24.082	0.0561
	Null	13	42,478	
OmtA-10	Transgenic	12	34,731	0.5085
	Null	5	27.486	
OmtA-11	Transgenic	18	33,455	0.6656
	Null	6	43,365	
OmtA-12	Transgenic	19	29,785	0.0635

Table S2. Primers and probes used for genotyping and zygosity test of OmtA-RNAi events

Primer name	Oligonucleotide sequence (5′→3′)
OmtA-F	CGACTTGCTTGGGTCCAT
OmtA-R	AAGATCGGGCATAATCATTTC
OmtA-F (RT-PCR)	AGGAGGTTGCCCCTGATCA
OmtA-R (RT-PCR)	TGCCCGTTACTCGCAACAT
OmtA-F (Taq)	CGG AGT TGA GGA CAC TGA TAA A
OmtA-R (Taq)	CAG CAT CGG GAT AGT CAT GTA G
aattB4-OmtA-F	*GGGACAACTTTGTATAGAAAAGTT*ATCTAGGTATGGGTGGCACC
aattB1-OmtA-R	*GGGACAACTTTGTATAGAAAAGTT*AAGATCGGGCATTTC
aattB2-OmtA-F	*GGGACAACTTTGTATAGAAAAGTT*CGACTTGCTTGGGTCCAT
aattB3-OmtA-F	*GGGACAACTTTGTATAGAAAAGTT*ATCTAGGTATGGGTGGCACC
OmtA-probe*	FAM/ATC GGA GAA/ZEN/GAT AGA CAT CGG CGC/3IABkFQ
Adh-F (RT-PCR)	CGTCGTTTCCCATCTCTTCCTCC
Adh-R(RT-PCR)	CCACTCCGAGACCCTCAGTC
Adh Probe*	HEX-AATCAGGGCTCATTTCTCGCTCCTCA-IBFQ
Bar-F	TGCACCATCGTCAACCACTACATCGAG
Bar-R	CAGGTGAAGTCCAGCTGCCAGAAAC
Bar-F (Taq)	GGA AGT TGA CCG TGC TTG T
Bar-R (Taq)	GAT CTA CCA TGA GCC CAG AAC
Bar-probe*	FAM-CGATGTAGT/ZEN/GGTTGACGATGGTGCA-IBFQ

Italics indicate the homologous recombination sites that is added to the end of the gene specific primer sequences. *: the probes were labeled with FAM (6-fluorescein) or HEX (hexachloro fluorescein) at the 5′end and quenched with Iowa Black FQ (IBFQ) or ZEN and IBFQ.

Table S3. List of RNAi-OmtA lines from 12 transformation events received from ISU after transformation

Event	Total number of Kernels	Kernel weight (g)
OmtA-1	78	0.1794
OmtA-2	27	0.2146
OmtA-3	6	0.1657
OmtA-5	142	0.1500
OmtA-6	50	0.2459
OmtA-7	86	0.2057
OmtA-8	66	0.2317
OmtA-9	50	0.2599
OmtA-10	95	0.1705
OmtA-11	120	0.1750
OmtA-12	28	0.2034

Table S4: Information on the self-pollination of OmtA lines from 2017 to 2019 and crosses with three elite lines produced in 2018

Year	Lines	# of seeds	# of seedlings	# of transgenic	# of plants pollinated	# of ears inoculated[$]	Total # of ears harvested
2017 selfing	OmtA-7	160	131	44	35	12	34
(T3 to T4)	Null-7	60	48	N/A	45	12	37
	OmtA-12	150	120	44	40	10	36
	Null-12	80	70	N/A	58	11	50
	B104-13	100	61	N/A	54	7	23
2018 selfing	OmtA-7	180	174	174	65	12	63
(T4 to T5)	Null-4	150	143	N/A	83	11	75
and F1	OmtA-12	158	106	106	67	9	35
crossing	Null-12	85	78	N/A	52	10	48
	B104-9	100	87	N/A	57	14	44
	LH195	206	169	N/A	39*	23*	36*
	PHN46	197	174	N/A	40*	29*	36*
	PHG39	210	180	N/A	35*	28*	33*
	OmtA-7		50				
	Null-7		50				
2019 selfing	OmtA-7	75	49	49	≥40	15	>25
T5 to T6	Null-7	75	51	N/A	≥40	15	>25
(OmtA-7	OmtA-12	75	53	53	≥40	7	>25
and OmtA-	Null-12	75	55	N/A	≥40	10	>25
12) and T4	OmtA-6	150	125	38	35	9	33
(OmtA-6	Null-6	60	55	N/A	49	7	45
and OmtA-	OmtA-10	150	122	34	37	13	30
10)	Null-10	60	58	N/A	56	13	51

$ Number of ears inoculated at 2 weeks after pollination.
* Among these number of inbred plants, 1/3 were crossed with homozygous OmtA-7, 1/3 of them were with null OmtA-7, and the remaining 1/3 were self-pollinated as control.

Chen, Z.-Y., Brown, R.L., Damann, K.E., and Cleveland, T.E. (2010). PR10 expression in maize and its effect on host resistance against *Aspergillus flavus* infection and aflatoxin production. Molecular Plant Pathology **11:** 69-81.
